# Controlled, cross-sectional, multi-center study of physical capacity and associated factors in women with fibromyalgia

**DOI:** 10.1186/s12891-018-2047-1

**Published:** 2018-04-19

**Authors:** Anette Larsson, Annie Palstam, Jan Bjersing, Monika Löfgren, Malin Ernberg, Eva Kosek, Björn Gerdle, Kaisa Mannerkorpi

**Affiliations:** 1 Institute of Neuroscience and Physiology, Section of Health and Rehabilitation, Physiotherapy, Sahlgrenska Academy, University of Gothenburg, Box 430, 40530 Gothenburg, Sweden; 2 University of Gothenburg Centre for Person-Centred Care (GPCC), Gothenburg, Sweden; 3 Institute of Neuroscience and Physiology, Department of Clinical Neuroscience, Sahlgrenska Academy, University of Gothenburg, Gothenburg, Sweden; 4 Department of Rheumatology, Sahlgrenska University Hospital, Gothenburg, Sweden; 5 Department of Clinical Sciences, Karolinska Institutet and Department of Rehabilitation Medicine, Danderyd Hospital, Stockholm, Sweden; 6 Department of Dental Medicine, Karolinska Institutet, and Scandinavian Center for Orofacial Neurosciences (SCON), Huddinge, Sweden; 7 Department of Clinical Neuroscience, Karolinska Institute and Stockholm Spine Center, Stockholm, Sweden; 8 Pain and Rehabilitation Centre, Department of Medical and Health Sciences, Linköping University, Linköping, Sweden

**Keywords:** Muscle strength, Fibromyalgia, Physical capacity, Symptom duration, Activity limitations, Walking ability

## Abstract

**Background:**

Health and physical capacity are commonly associated with disease, age, and socioeconomic factors. The primary objective of this study was to investigate the degree to which physical capacity, defined as muscle strength and walking ability, is decreased in women with fibromyalgia (FM), as compared to healthy women, who are matched for age and level of education. The secondary aim was to investigate whether muscle strength and walking ability are associated with age, symptom duration, activity limitations and, Body Mass Index (BMI) in women with FM and control subjects.

**Methods:**

This controlled, cross-sectional, multi-center study comprised 118 women with FM and 93 age- and education-level-matched healthy women. The outcome measures were isometric knee-extension force, isometric elbow-flexion force, isometric hand-grip force, and walking ability. Differences between the groups were calculated, and for the women with FM analyses of correlations between the measures of physical capacity and variables were performed.

**Results:**

The women with FM showed 20% (*p* < 0.001) lower isometric knee-extension force, 36% (*p* < 0.001) lower isometric elbow-flexion force, 34% (*p* < 0.001) lower isometric hand-grip force, and 16% lower walking ability (*p* < 0.001), as compared to the healthy controls. All measures of muscle strength in women with FM showed significant weak to moderate relationship to symptom duration (r_s_ = − 0.23–0.32) and walking ability (r_s_ = 0.25–0.36). Isometric knee-extension force correlated with activity limitations, as measured using the SF-36 *Physical function* subscale (r_s=_0.23, *p* = 0.011).

**Conclusions:**

Physical capacity was considerably decreased in the women with FM, as compared to the age- and education-level-matched control group. All measures of physical capacity showed a significant association with symptom duration. Knee-extension force and walking ability were significantly associated with activity limitations, age, and BMI. It seems important to address this problem and to target interventions to prevent decline in muscle strength. Assessments of muscle strength and walking ability are easy to administer and should be routinely carried out in the clinical setting for women with FM.

**Trial registration:**

ClinicalTrials.gov identification number: NCT01226784, Oct 21, 2010.

## Background

Fibromyalgia (FM) is a musculoskeletal pain condition that affects approximately 1–3% of the general population, it is more common among women and prevalence increases with higher age [1, 2]. FM is characterized by persistent widespread pain, increased pain sensitivity and tenderness [3] and is often associated with impaired physical capacity [4–6], activity limitations [7], fatigue, and distress [3, 8]. While the etiology of FM is still poorly understood it is generally agreed that disturbances in both the central and peripheral pain modulating mechanisms contribute to the maintenance of widespread pain [9, 10].

Physical capacity, which includes aerobic capacity, muscle strength, flexibility and balance, influences the ability to manage tasks of everyday living [11]. Muscle strength is essential for performing activities, such as walking stairs, lifting and carrying heavy objects, and rising from a chair. To date, descriptions of muscle strength have been based mainly on patients examined in a specialist rheumatology setting, and the results show that muscle strength in women with FM is reduced by 20%–35% [5, 12–14], as compared to healthy women. Maquet et al. [13] compared 16 women with FM to 85 healthy controls and found that knee-extension strength was on average reduced by 35% in the women with FM. Norregaard et al. [15] investigated 181 women with FM and 126 healthy women and found that isokinetic muscle strength in the flexors and extensors of the knee and elbow was reduced by 20%–30% in the women with FM. Cardoso el al [14], in a relatively small study, found significantly reduced strength levels related to isokinetic knee flexion and extension in patients with FM. Henriksen et al. [5] compared 844 women with FM, and 122 healthy controls and found that isokinetic knee extension strength was reduced by 15%–25% in the women with FM. However, half of the patients in this sample had near-normal levels of muscle strength [5].

Possible physiologic explanations for the reduced muscle strength include pathologic changes in muscle fibres [16], impaired blood circulation [17], and disturbances in regulation of growth and energy metabolism [18]. Other possible explanations include altered neuromuscular control mechanisms caused by pain [19] or decreased levels of physical activity in women with FM [20]. Women with FM spend more time engaging in sedentary activities and less time engaging in high-intensity activities [21].

High muscle strength has been associated with lower levels of pain in persons with FM [22]. No studies have, to our knowledge, investigated the association between pain duration and muscle strength in women with FM.

Muscle strength also decreases with increasing age due to changes in body composition, resulting in increased fat mass and age-related loss of muscle mass. This age-related loss of muscle mass leads to reduced strength and as a consequence decreased physical capacity [23]. Being overweight or obese, which is often the case in persons with FM [24], has also been associated with systemic low-grade chronic inflammation [25]. There appears to be an increased risk of developing or augmenting FM symptoms when one is overweight or obese, especially for women who report low levels of physical activity [26].

In addition socioeconomic factors, such as education level, have a strong positive association to health and physical capacity [27]. The odds of having FM are strongly linked to having poor education-level [8, 28]. Well-educated people in the general population report better health and physical capacities and are more likely to engage in positive health behaviors such as physical activity [27]. However, to the best of our knowledge, no previous study of the physical capacity in women with FM have controlled for education level in combination with age.

### Aims

The primary aim of this study was to investigate the degrees to which muscle strength and walking ability are decreased in women with FM, as compared to healthy women, who are matched for age and level of education. A secondary aim was to investigate whether muscle strength and walking ability are associated with the age, symptom duration, activity limitations, and Body Mass Index (BMI) of women with FM and in healthy women.

## Methods

### Study design

This is a controlled, cross-sectional, multi-center study.

This is a sub-study of a multi-center experimental study that enrolled women with FM and healthy women (ClinicalTrials.gov identification number: NCT01226784). The recruitment started in Year 2010 and data collection was completed at all sites in Year 2013. The outcome measures were isometric knee-extension force; isometric elbow-flexion force; isometric hand-grip force; walking ability; and activity limitations.

### Participants

Both the women with FM and the healthy controls were recruited through newspaper advertisements in the local daily newspapers of Gothenburg, Stockholm, and Linköping [29].

The inclusion criteria for the women with FM were: to be within the age range of 20–65 years; and meeting the American College of Rheumatology (ACR) 1990 classification criteria for FM [3]. Exclusion criteria included: high blood pressure (> 160/90 mmHg); osteoarthritis (OA) of the hip or knee, confirmed by radiologic findings; other severe somatic or psychiatric disorders; dominating causes of pain other than FM; participation in a rehabilitation program or regular resistance exercise; and inability to understand or speak Swedish. In total, 402 women with FM who declared their interest in participating in the study were screened in a telephone interview for eligibility and informed about the study procedure. Out of these, 177 women who were interested in participating were referred to a medical examination in which they were screened for eligibility. Overall 130 women with FM within the age range of 22–64 years fulfilled the inclusion criteria and formed the cohort of the study. Matched controls were recruited in parallel from a cohort of 130 healthy women within the age range of 21–64 years. For the latter group the exclusion criteria were: any pain condition; high blood pressure (> 160/90 mmHg); OA of the hip or knee; and other severe somatic or psychiatric disorders.

The patients and healthy controls were individually matched according to age and education. Initially, the participants were classified into 5-year age groups, while the education categories were based on the exact number of years of education received. After this matching process, additional patients in the education level 10–12 were included. The final study sample comprised 118 women with FM and 93 healthy women. The difference in mean age between the two groups was only 0.2 years (*p* = 0.91) and the percentage difference in education level was less than 15% (*p* = 0.33), Table 1.

**Table 1:** Characteristics of study participants

	Women with FM (*n* = 118)	Healthy women (*n* = 93)	
Characteristics	Mean(SD)	Mean(SD)	*p*-value
Age(years)	51.04 (9.51)	51.19 (9.64)	0.910
Symptom duration (years)	10.16 (7.95)	n.a	n.a
Tenderpoints (number)	15.6 (1.99)	n.a	n.a
Current pain (0–100)	50.5 (21.16)	2.98 (6.62)	< 0.001
FIQ total (0–100)	60.4 (15.6)	6.3 (8.7)	< 0.001
LTPAI (hours)	5.71 (5.64)	6.98 (4.62)	0.001
Body Mass Index (kg/m^2^)	27.90 (5.28)	24.70 (3.48)	< 0.001
	n (%)	n (%)	
Education			
≤9 years	11 (9%)	11 (12%)	
10–12 years	56 (48%)	31 (33%)	
> 12 years	51 (43%)	51 (55%)	0.102
Living with an adult Yes	77 (65%)	73 (79%)	0.251
Born in Sweden Yes	106 (90%)	83 (89%)	0.891
Pharmacological treatment			
NSAID, paracetamol *Yes*	88 (75%)		
Opioids for mild to moderate pain *Yes*	13 (11%)		
Antidepressants *Yes*	52 (44%)		
Anticonvulsives *Yes*	5 (4%)		
Sedatives *Yes*	22 (19%)		

*FIQ* Fibromyalgia Impact Questionnaire, *LTPAI* Leisure Time Physical Activity Index, *NSAID* non-steroidal anti-inflammatory drug

Missing: healthy women living with an adult (n = 1); FIQ total healthy women (*n* = 3)

### Data collection

Background data were collected through interviews and self-reported questionnaires. The collected data included age, symptom duration, education (*≤9 years, 10–12 years, > 12 years*), family status (*living with an adult, yes/no*), and country of birth (*born in Sweden, yes/no*). Current pain was assessed on the Visual Analogue Scale (VAS). The participants also completed the Fibromyalgia Impact Questionnaire (FIQ total) [30], which is a disease-specific instrument, and the Leisure Time Physical Activity (LTPAI) [31] which is an instrument that assesses the amount of physical activity performed during a typical week (h). The participants were also asked about pharmacological treatments (*NSAIDs, Paracetamol yes/no, Opioids for mild to moderate pain yes/no, Antidepressants yes/no, Anticonvulsives yes/no, Sedatives yes/no*). Height and weight were measured and BMI [*weight (kg) / height* (m)^2^] was calculated. Clinical assessment of tender points was carried out by manual palpation [3]. Examinations were conducted at physiotherapy clinics in Gothenburg, Linköping, and Stockholm. Performance-based tests of muscle strength and walking ability are presented below in the order in which they were performed.

#### Measures of muscle strength and walking ability

##### Muscle strength

Maximal isometric knee-extension force (N) was measured with a dynamometer *(Steve Strong®; Stig Starke HBI, Göteborg, Sweden)*. The participant was set in a seated position, knee and hip in 90 ° of flexion and back supported. The subjects were verbally encouraged to pull the ankle strap with maximal force for 5 s, and between each trial there was a 1-min rest interval. The best performance out of three trials was recorded. A mean value from the right and left leg was calculated. This measure has been applied previously [32, 33], and has shown high test-retest reliability for patients with a chronic disease [33]. Maximal isometric elbow-flexion force (N) in both arms, assessed one by one, was measured using a dynamometer *(Isobex®; Medical Device Solutions AG, Oberburg, Switzerland)*. The participant was sitting without back support with the upper arm aligned with the trunk. The elbow was placed in 90° of flexion, forearm supinated. The maximum strength preformed during a period of 5 s was recorded. Three trials were performed for each arm and between each trial there was a 1-min rest interval [34]. The best performance out of the three trials was recorded. A mean value from the right and left arm was calculated. Hand-grip force (N) bilaterally was assessed using the *Grippit®* device *(AB Detektor, Göteborg, Sweden)*. Two trials were performed for each hand with one minute rest interval between each trial and the mean force over a set period of time (10 s) was recorded [35]. The best performance out of the two trials was recorded. A mean value of the right and left hand was calculated and used in the present study. *Walking ability:* Walking ability was assessed with the Six-Minute Walk Test (*6MWT*)*,* which is a performance-based test measuring total walking distance (m) [36]. The participant was instructed to walk as fast as possible without running for a period of 6 min. The total distance covered in meters was recorded. The test was performed along a 30-m-long, straight corridor, turnaround points marked with a cone. The 6MWT is considered a useful representation of physical capacity and endurance in daily life.

#### Measures of activity limitations

##### Activity limitations

Activity limitations were measured using the Short-Form Health Survey (*SF-36*) *Physical function* subscale [37], which includes questions about vigorous activities such as lifting and carrying heavy objects, climbing stairs and walking several blocks. A higher score indicates fewer activity limitations, i.e., good physical function.

### Statistical analysis

The data were analyzed using the Statistical Package Software for the Social Sciences (SPSS ver. 22.0; SPSS, Armonk, NY, USA). Descriptive data are presented in the forms of mean and standard deviation (SD), for continuous variables or the number (n) and percentage (%) for categorical variables. All significance tests were two-sided and conducted at the 5% significance level. Differences between groups were calculated with the Mann-Whitney U-test for continuous variables and with Fisher’s exact-test for dichotomous variables. Spearman’s rank correlation coefficient was used for analyzing correlations between variables. The following classification system was used to interpret the absolute (irrespective of sign) correlation values, given that the *p*-values were ≤ 0.05: *r*s 0.01–0.25 indicates a weak relationship, *r*s 0.25–0.50 indicates a moderate-level relationship, *r*s 0.50–0.75 a moderate-to-good relationship, and above *r*s 0.75 a very good-to-excellent relationship [38].

## Results

The characteristics of the participants are presented in Table 1.

There were no significant differences between the women with FM and the healthy women regarding age, level of education, living with an adult or being born in Sweden. The women with FM displayed significantly higher pain levels, activity limitations, and BMI and significantly lower leisure-time physical activity levels than the healthy women. Seventy-five percent of the women with FM reported the use of non-steroidal anti-inflammatory drugs (NSAID) or paracetamol, 11% reported use of opioids, 44% reported use of antidepressants, 4% reported use of anticonvulsants and 19% reported use of sedatives, Table 1.

### Physical capacity, (assessed as muscle strength and walking ability), in women with FM versus healthy controls

The women with FM showed significantly lower isometric knee-extension force (*p* < 0.001), isometric elbow flexion force (*p* < 0.001), isometric hand-grip force (*p* < 0.001), and walking ability (*p* < 0.001) than the healthy women, Table 2. The women with FM had 20% lower isometric knee-extension force, 36% lower isometric elbow-flexion force, and 34% lower isometric hand-grip force than the healthy women. They also walked 16% shorter distances in the 6MWT than the healthy controls.

**Table 2:** Differences in muscle strength and walking ability between women with FM and healthy women

	Women with FM (*n* = 118)	Healthy women (*n* = 93)	
	Mean (SD)	Mean (SD)	*p*-value
Isometric knee-extension force (N)	322.8 (108.9)	401.3 (81.7)	< 0.001
Isometric elbow-flexion force (N)	120.1 (49.3)	185.3 (52.5)	< 0.001
Hand-grip force (N)	152.8 (65.3)	233.3 (56.9)	< 0.001
6MWT (m)	550.1 (72.5)	648.8 (64.4)	< 0.001

*6MWT* Six Minute Walk Test

### Intercorrelations between physical capacity variables

The investigated physical capacity variables intercorrelated significantly for the women with FM (r_s_:0.25–0.75; Table 3) and for the healthy controls (r_s_: 0.44–0.74; Table 4).

**Table 3:** Correlations between muscle strength, walking ability, and variables in women with FM (*n* = 118)

Variables	Isometric knee extension force (N)	Isometric elbow flexion force (N)	Hand-grip force (N)	6MWT (m)
	r_s_ (*p*-value)	r_s_ (*p*-value)	r_s_ (*p*-value)	
Isometric knee extension force (N)	–	0.72 (< 0.001)	0.61 (< 0.001)	0.36 (< 0.001)
Isometric elbow flexion force (N)	0.72 (< 0.001)	–	0.75 (< 0.001)	0.25 (0.006)
Hand-grip force (N)	0.61 (< 0.001)	0.75 (< 0.001)	–	0.34 (< 0.001)
6MWT (m)	0.36 (< 0.001)	0.25 (0.006)	0.34 (< 0.001)	–
Age (years)	−0.19 (0.043)	−0.041 (0.656)	−0.10 (0.276)	− 0.24 (0.009)
BMI	0.19 (0.039)	0.03 (0.792)	− 0.05 (0.589)	− 0.25 (0.007)
Symptom duration (years)	− 0.28 (0.002)	− 0.32 (< 0.001)	− 0.23 (0.012)	− 0.26 (0.004)
SF36 PF (0–100)	0.23 (0.011)	0.11 (0.23)	0.16 (0.089)	0.42 (< 0.001)

*6MWT* Six Minute Walk Test, *BMI* Body Mass Index, *SF36 PF* Short Form 36 physical function subscale

**Table 4:** Correlations between muscle strength and independent variables in healthy women (*n* = 93)

Independent variables	Isometric knee extension force (N)	Isometric elbow flexion force (N)	Hand-grip force (N)	6MWT (m)
	r_s_ (*p*-value)	r_s_ (*p*-value)	r_s_ (*p*-value)	
Isometric knee extension force (N)	–	0.62 (< 0.001)	0.60 (< 0.001)	0.33 (0.001)
Isometric elbow flexion force (N)	0.62 (< 0.001)	–	0.74 (< 0.001)	0.40 (< 0.001)
Hand-grip force (N)	0.61 (< 0.001)	0.74 (< 0.001)	–	0.44 (< 0.001)
6MWT (m)	0.33 (0.001)	0.40 (< 0.001)	0.44 (< 0.001)	–
Age (years)	− 0.28 (0.007)	− 0.15 (0.163)	− 0.23 (0.025)	− 0.29 (0.005)
BMI	− 0.07 (0.538)	0.24 (0.023)	− 0.18 (0.086)	− 0.41 (< 0.001)
SF36 PF (0–100)	0.14 (0.170)	0.21 (0.047)	0.19 (0.065)	0.33 (0.001)

*6MWT* Six Minute Walk Test, *BMI* Body Mass Index, *SF36 PF* Short Form 36 physical function subscale

#### Correlations between physical capacity, (assessed as muscle strength and walking ability), age, BMI, symptom duration, and activity limitations for women with FM

Both isometric knee extension force and 6MWT correlated significantly with age, BMI, symptom duration and SF36 *Physical function* (Table 3); the strength of the correlations was weak to moderate. Isometric elbow-flexion force and hand-grip force correlated with symptom duration (Table 3). The strength of the correlations was weak to moderate.

#### Correlations between physical capacity, (assessed as muscle strength and walking ability), age, BMI, and activity limitations for healthy women

The correlation coefficients are presented in Table 4. 6MWT correlated significantly with age, BMI, and SF36 physical function (Table 4); the strength of the correlations was moderate.

## Discussion

Muscle strength in women with FM was found to be 20%–36% lower and walking ability was 16% lower than the corresponding levels for healthy women.

As age and socioeconomic factors, such as education level, have been linked to health status, our FM sample was compared to a group of healthy women, who were matched for age and level of education. One reason for the differences in health variables in different socioeconomic groups may be that, well-educated persons are more likely to exercise and to follow a healthy lifestyle [27]. Furthermore, previous studies have shown an association between higher presence of FM and low levels of education [8, 28]. Although the groups in the present study were matched, there were considerable differences in physical capacity, especially regarding muscle strength, between the two groups. This implies that there are disease-related physiologic or neuromuscular factors that affect the physical capacity of women with fibromyalgia.

Both the women with FM and the healthy controls were recruited from the general population through advertisements, and matched according to age and level of education. Our results showing decreased physical capacity are in line with the results of previous studies that were based on examinations of patients recruited from specialist clinics [5, 12–14] but not matched for age and education.

Symptom duration in women with FM was associated with reduced physical capacity for all four variables. This implies that disease-related factors, such as duration of pain, probably contribute to the decrease of physical capacity in women with FM. Although physical capacity decreases with age, it seems that a longer duration of FM symptoms further deteriorates physical capacity. That symptom duration affects physical capacity is an important thing to know for clinicians, as a previous study has shown that symptom duration is a predictor of falls in women with FM, probably due to reduced lower limb muscle strength [6].

The women with FM in the present study displayed 20% lower knee-extension force than the healthy women. Both knee-extension force and walking ability were associated with SF36 Physical function subscale which is as expected given that this subscale covers items in daily activities, such as walking stairs and walking several blocks. It is reasonable to assume that these types of activity limitations are associated with impairments of muscle strength and walking ability. Knee-extension force showed a moderate association with walking ability, a finding that is in line with previous studies [39, 40]. Reduced lower limb muscle strength, especially knee-extensor strength, has been shown to be associated with impaired balance [40] increasing the risk for falls [41], and also associated with problems with walking ability and balance in women with FM; a high incidence of falls (50%) has been reported in this population [24]. Walking ability has also been shown to be a predictor for work ability in patients with low back pain [42] .

Regarding upper body strength, the women with FM displayed 36% lower elbow-flexion force and 34% lower hand-grip force than the healthy women. Hand-grip force has been shown to be a good predictor of hospitalization and mortality and is useful for predicting activity limitations [43]. Low muscle strength has also been proposed to affect directly mortality through its association with increased disability [44]. The decline in muscle strength appeared to be more pronounced in the upper extremities than in the lower extremities.

The different measures of muscle strength were positively associated; implying that a decline in strength in one muscle group is associated to a decline in other muscle groups. This has been shown also in a previous study, in which hand-grip strength was shown to be strongly correlated to knee-extension strength in healthy adults [45]. The measures of muscle strength and walking ability were in line with those in previous studies that comprised healthy Swedish women [35, 36]. Three out of four measures of physical capacity in the healthy controls showed a small but yet significant association with age. In the healthy group, walking ability showed a significant association with BMI and activity limitations, implying a pattern similar to that seen for the women with FM.

Muscle-strengthening activity, such as resistance exercise, is recommended for the general population to promote health and physical independence [46], as well as to prevent the development of degenerative, age-related, chronic conditions [47]. The prevention of loss of muscle mass and physical function might be even more important for women with FM given their impaired muscle strength. Muscle strength can be affected through targeted interventions, and improving muscle strength by engaging in resistance exercise improves walking ability and reduces the risk for falls [41]. Patients with FM report activity limitations in daily life, and it seems important to address this problem and to target interventions to prevent a decline in muscle strength.

There are some limitations to this study. The recruitment procedure through newspaper advertisement may have biased the process of recruitment of participants. To minimize this risk, participants in the FM-group and in the healthy control group were recruited in a similar fashion. The study only comprised women since fibromyalgia is more common among women and the results cannot be generalized to men with fibromyalgia.

## Conclusion

Muscle strength and walking ability are considerably decreased in women with FM, as compared to healthy women of similar age and education level. It seems important to target interventions for women with FM that prevent these declines in their physical capacity. Assessment of muscle strength and walking ability are easy to administer and it is recommended that they be routinely assessed in the clinical setting. The results of the present study add to the body of knowledge that muscle function is an important component of health and disease.

## Abbreviations

6MWT: Six-Minute Walk Test
ACR: American College of Rheumatology
BMI: Body Mass Index
FIQ: Fibromyalgia Impact Questionnaire
FM: Fibromyalgia
LTPAI: Leisure Time Physical Activity Index
NSAID: Non-Steroidal Anti-Inflammatory Drug
SD: Standard Deviation
SF36: Short-Form Health Survey 36
